# A Qualitative Study Exploring Barriers Related to Use of Footwear in Rural Highland Ethiopia: Implications for Neglected Tropical Disease Control

**DOI:** 10.1371/journal.pntd.0002199

**Published:** 2013-04-25

**Authors:** Desta Ayode, Colleen M. McBride, Hendrik D. de Heer, Emi Watanabe, Tsega Gebreyesus, Abebayehu Tora, Getnet Tadele, Gail Davey

**Affiliations:** 1 College of Social Sciences, Addis Ababa University, Addis Ababa, Ethiopia; 2 Social & Behavioral Research Branch, National Human Genome Research Institute, National Institutes of Health, Bethesda, Maryland, United States of America; 3 Northern Arizona University, Department of Physical Therapy and Athletic Training, Flagstaff, Arizona, United States of America; 4 Department of International Health, Johns Hopkins Bloomberg School of Public Health, Baltimore, Maryland, United States of America; 5 Department of Sociology, Wolaita Sodo University, Sodo, Ethiopia; 6 Brighton and Sussex Medical School, Falmer, Brighton, United Kingdom; Dodowa Health Research Centre, Ghana

## Abstract

**Background:**

The role of footwear in protection against a range of Neglected Tropical Diseases (NTDs) is gaining increasing attention. Better understanding of the behaviors that influence use of footwear will lead to improved ability to measure shoe use and will be important for those implementing footwear programs.

**Methodology/Principal Findings:**

Using the PRECEDE-PROCEED model we assessed social, behavioral, environmental, educational and ecological needs influencing whether and when children wear shoes in a rural highland Ethiopian community endemic for podoconiosis. Information was gathered from 242 respondents using focus groups, semi-structured interviews and extended case studies. Shoe-wearing norms were said to be changing, with going barefoot increasingly seen as ‘shameful’. Shoes were thought to confer dignity as well as protection against injury and cold. However, many practical and social barriers prevented the desire to wear shoes from being translated into practice. Limited financial resources meant that people were neither able to purchase more than one pair of shoes to ensure their longevity nor afford shoes of the preferred quality. As a result of this limited access, shoes were typically preserved for special occasions and might not be provided for children until they reached a certain age. While some barriers (for example fit of shoe and fear of labeling through use of a certain type of shoe) may be applicable only to certain diseases, underlying structural level barriers related to poverty (for example price, quality, unsuitability for daily activities and low risk perception) are likely to be relevant to a range of NTDs.

**Conclusions/Significance:**

Using well established conceptual models of health behavior adoption, we identified several barriers to shoe wearing that are amenable to intervention and which we anticipate will be of benefit to those considering NTD prevention through shoe distribution.

## Author Summary

Consistently wearing shoes may help in preventing onset or progression of a wide range of Neglected Tropical Diseases (NTDs). This study assessed the factors that influenced shoe wearing behaviors among people living in a rural community in highland Ethiopia. In this community, a substantial proportion of people are at risk for podoconiosis, a debilitating lower leg condition that can be prevented by wearing shoes. We conducted semi-structured individual interviews, focus group discussions and extended case studies among 242 adults and systematically analyzed the information. We found that shoe wearing is intermittent, and that different factors such as cost and ability to use the shoes for certain activities (such as farming) influenced consistent shoe wearing for most people. Some factors (such as shoe size, fear of stigma) were more relevant for podoconiosis patients. Social norms were found to be increasingly supportive of shoe wearing, and children exhibited greater desire to wear shoes than adults. These findings have relevance for preventing development and progression of a variety of NTDs in a range of settings.

## Introduction

Interest is growing in the use of footwear in the primary prevention of certain Neglected Tropical Diseases (NTDs). While evidence for a protective role of footwear against podoconiosis [1]–[3] and chronic larva migrans [4], [5] is relatively strong, evidence for the role of shoes is inconsistent in relation to hookworm, with some studies finding evidence of protection from footwear [6], [7], but other studies finding no effect [8]–[11]. Evidence is also inconsistent for other helminthiases [10], [12]–[15] and Buruli ulcer [16], [17]. For snakebite and tungiasis, evidence of protection is circumstantial, and based on the predilection of bites [18] and lesions [19] for the feet.

While research on the impact of behaviors such as hand-washing [20], face-washing [21] and use of disease-preventing commodities such as insecticide-treated bed nets (ITNs, [22]) is relatively advanced, there is a paucity of research on behaviors related to footwear and their impact on NTDs.

While conducting work on the use of shoes in a rural Ethiopian community endemic for podoconiosis (a NTD triggered by exposure to irritant soils in the tropical highlands [3], [23]), we uncovered considerable information on behaviors and practices relating to shoe use which is relevant to a range of other NTDs. In brief, in southern Ethiopia, shoes are being distributed through a local non-governmental organization to children with the intention of preventing podoconiosis. This non-communicable form of elephantiasis arises from long-term exposure to red clay soils. Ecological and observational evidence suggests that consistent use of shoes prevents disease by protection from soil exposure. Shoe distribution to children of treated patients has been accompanied by messages linking foot hygiene and shoe use to reduced risk of disease. Program implementers considered it vital to understand why children might or might not wear shoes, in order to improve the messaging that might be used alongside distribution. To this end, we drew on several conceptual models to guide our efforts. First, we relied on the PRECEDE-PROCEED model that suggests beginning the process with diagnostic planning to assess social, behavioral, environmental, educational and ecological issues and needs that may influence whether and when children wear shoes [24]. We also considered social cognitive theory of self regulation [25]. Taken together these theories argue for the importance of targeting individuals' beliefs and attitudes about shoe wearing, how these beliefs influence perceived capabilities to prevent podoconiosis, and whether wearing shoes can be effective in reducing their risk for the condition. The data presented in this article arise from a qualitative study aimed to gain deeper understanding of the barriers to consistent use of shoes in a rural setting.

We anticipate that this information will be valuable both for investigators designing future studies to assess the association between shoe use and incidence of NTDs, and for those developing shoe-related prevention programs.

## Methods

### Ethics statement

Ethical approval was granted by the Institutional Review Boards of Addis Ababa University Medical Faculty and the National Human Genome Research Institute, National Institutes of Health, USA. Oral consent was obtained from all study participants by a trained research assistant, following the procedures developed and evaluated by Tekola and colleagues using Rapid Ethical Assessment in this community [26]. In brief, Rapid Ethical Assessment is a form of rapid anthropological assessment performed to explore a community's understanding of research, to document how a community prefers to be approached by investigators and to detail how community members wish consent to be given. Rural communities in Wolaita prefer contact through a Mossy Foot Treatment and Prevention Association (MFTPA) staff member prior to individual discussion and consent. The Mossy Foot Treatment and Prevention Association is a local non-governmental organization involved in the prevention and treatment of podoconiosis patients and shoe distribution for their children. Oral consent is preferred by this community [26], was approved by the IRBs mentioned above and was documented by a witness on each occasion following an explanation of the research protocol using the information sheet and consent form.

### Study setting and design

The study was conducted in Wolaita zone in southern Ethiopia, where the population is estimated to be 1.7 million [27]. Podoconiosis is known to be prevalent in this zone [28]. Most of the villagers are subsistence farmers. The study was entirely qualitative and employed multiple methods (focus group discussion (FGD), in-depth interviews (IDI) and case studies) to gain an in-depth understanding of community perspectives on behaviors related to shoe use, and the predominant facilitators and barriers to wearing shoes. Structured topic guides were used to direct discussions, focusing on local explanations for the causes of podoconiosis, attitudes towards individuals affected by podoconiosis, attitudes towards wearing shoes, and optimal role models and settings for promoting footwear among high-risk children. Case studies enabled deeper and more contextualized information to be gathered around an individual, with information gathered from the individual, from family members and from friends. An article describing community perceptions surrounding risk factors and prevention, including how adults' explanations of disease heredity influence shoe wearing and interpersonal behaviors, has recently been published [29]. The present paper focuses on the perceptions of participants regarding footwear and explores the major factors impeding shoe use in the community.

### Sampling

Participants were recruited using convenience and snowball sampling methods. A total of 242 adults participated from the following three groups: (1) 69 adults affected with and receiving treatment for podoconiosis, “affected”; (2) 129 unaffected adults, with no current sign of or previous history of podoconiosis, “unaffected”; and (3) 44 community and religious leaders “community and religious leaders”. None of the community leaders and religious leaders was currently affected by disease. The study took place in four of 14 communities served by the Mossy Foot Treatment and Prevention Association (MFTPA) a local non-governmental organization involved in the prevention and treatment of podoconiosis patients and shoe distribution for their children. The four sites were selected to represent the diversity of communities served with respect to size, duration of the relationship with MFTPA, and distance from the main office of the MFTPA.

### Data collection

This study was conducted from June to August 2010. The month of June was partly dry while the rest of the study was conducted in the rainy season. This allowed the researchers to observe community shoe wearing practices during both the dry and the rainy seasons. A trained research assistant (Desta Ayode - DA) spent up to three weeks in each of the four communities with Abebayehu Tora (AT) and one other data collector conducting focus group discussions, semi-structured in-depth interviews and extended case studies with research participants. A total of 38 IDIs, 28 FGDs and 7 case studies were conducted in the study sites. All materials used for the study were developed in English, and then translated into Amharic and Wolatigna. The discussion and interviews were conducted in either Amharic or Wolatigna, and were audio-recorded. The audio-recordings were first transcribed in the language in which they were conducted (either in Amharic or Wolatigna), then translated into English. Translations of both study materials and transcripts were checked for consistency and to evaluate accuracy of important concepts.

### Data coding and analysis

A total of four coders with different backgrounds (Hendrik de Heer – the Netherlands, Emi Watanabe - Japan, Desta Ayode – Ethiopian resident and Tsega Gebreyesus – Ethiopian diaspora, the latter two Amharic speakers) were involved in developing the coding scheme and coding the data in order to maximize the breadth and depth of the analysis. After initial reading of the transcripts, the interview themes served as a starting point for the codebook, and subthemes were created as they emerged from the data. These overarching themes included barriers and advantages to wearing shoes, beliefs about podoconiosis and perspectives on best settings for interventions to facilitate shoe wearing as a means of prevention of podoconiosis and other diseases. In weekly meetings, any suggested categories or themes to add were discussed and agreed upon by all coders before being added to the list of themes and sub-themes. All coders coded multiple data sources and overlapped with each of the three other coders. Every inconsistency between coders for a given source (e.g. the transcript of a focus group) was resolved through discussion. The first 10% of all transcripts was coded by all four coders and two-thirds of all transcripts were coded by at least two coders. NVIVO-9 Qualitative data analysis software was used to assess all themes in the transcripts (NVIVO, QSR International, Burlington, MA 01803, USA). For example, the major sub-themes that emerged for barriers to consistent shoe-wearing included: i) financial barriers, ii) unsuitability of available shoes for certain activities, iii) low perceptions of adverse consequences as a result of not wearing shoes, iv) difficulty finding appropriate shoe sizes and v) fear of stigmatization as a result of wearing certain shoes. These themes are discussed in greater detail in the results section.

## Results

Results are presented in two main categories – barriers to consistent use of shoes, and community perceptions favoring footwear. Barriers to consistent use of shoes are further divided into four categories including those related to: 1) limited financial resources; 2) the unsuitability of shoes for specific activities; 3) a low perception of risk; 4) a fear of stigma. Quotes are attributed according to the age, gender and disease status of the participant.

### A. Barriers to consistent use of shoes

Although many respondents aspired to wear shoes, the reality was rather different. Many adults possessed shoes, and most parents stated that they were trying to buy shoes for their children; however, respondents observed that shoes were not worn regularly by most members of their community. As stated above, barriers faced by all community members included financial constraints, poor access to footwear appropriate to a range of local activities, and low perceptions of disease risk. Podoconiosis patients faced two additional barriers: difficulty finding large enough sizes, and fear of stigma and labeling.

#### i) Financial constraints

Participants at all the study sites repeatedly stated that financial issues were the main barriers to consistent use of shoes. Limited economic resources forced parents to focus on the most pressing priorities of feeding and educating their children rather than buying them shoes:

“I wish I could wear shoes everywhere, but the problem is lack of capacity to purchase shoes…tax, school expenses for children, clothes, and other expenses limit our capacity to buy shoes. Sometimes we are also heavily hit by drought.” (Unaffected, male, age 52)

“In spite of the fact that we know about the causes of the disease, we cannot afford to buy shoes regularly for ourselves or our children because of the dire poverty we are living. We might be able to buy a single pair once in a blue moon, but cannot replace it with a new pair every time the old pair is worn out” (Unaffected female, age 60)

Financial constraints act in a range of ways to cause inconsistent use of shoes. Several respondents said that consistently wearing a single pair of shoes would wear them out faster, whereas occasional use makes them last longer, meaning less frequent replacement.

“There are some individuals, who even though they have shoes, do not use them properly. In the interests of saving the shoes, some people carry their shoes on their shoulder and wear them when they arrive at the market place or in town and they do similarly when they come back home.” (Unaffected male, age 90)

Another response to financial limitations was to limit use of shoes to special occasions. Social occasions (weddings and funerals) and public places (churches, schools, and markets) were described by participants as settings in which most people wear shoes, whereas the home compound and farm fields were settings in which shoes were unlikely to be worn. Restricting the use of shoes to certain settings was linked to the wish to prolong the life of the shoes and the inability to buy a range of shoes for different settings.

“People cannot wear shoes everywhere due to lack of alternative pairs of shoes. They wear it economically only when they go to places like funerals, weddings, churches, market and other faraway places. They fear that the shoe might get old if they wear it in the farm, in the rain or performing other household activities. Some people even take off shoes while it is raining for fear it may destroy their shoes. …when going to town, some people take off their shoes if the road is muddy and put it on when they reach the dry roads”. (Community leader, male, age 50)

A further problem arises when price rather than quality is the major concern when buying shoes.

“I cannot dream of having quality shoes…I buy plastic shoes for myself and my children, spending about 12–16 birr [$US 1] each [pair]”. (Unaffected male, age 35)

The cheaper the shoe, the less durable it usually will be, requiring frequent replacement, which is challenging for most parents. People are likely to go barefoot once their shoes have worn out and before replacements can be bought –

“My children walk barefoot until I buy shoes for them next year” (Unaffected male, age 40)

Buying shoes for all children at once may be impossible for some large families, who therefore have to choose who should be the first to receive shoes. Some parents prioritize their older children, and even then, resort to a range of tactics to minimize wear and tear of the shoes.

“Sometimes we even hide the shoes from the children …” (Unaffected female, age 30)

As a result, scarcity of shoes may become a source of conflict, especially between children and parents. The following anecdotes vividly illustrate this -

“Even when we buy shoes for children, we don't let them wear them the whole time in order to preserve them. We punish the children when we find them wearing shoes at home. We force them to wear them only when they go to church, school and other distant places.” (Unaffected male, age 30)

“If I have five or six children, it is impossible to buy shoes for all of them. If I buy second hand shoes for the oldest child, I keep it secret from the rest of the siblings, as if he himself bought the shoes by selling a bunch of grass or firewood in the market. I hide them because the other siblings would be disappointed and would expect me to buy shoes for them.” (Community leader, male, age 50)

These responses suggest that the influence of parents on the shoe wearing behavior of children is critical: parents exert important influence over whether or not children adopt consistent shoe wearing habits in their early years.

Financial constraints often resulted in possession of only one pair of shoes, which for several reasons illustrated below contributed to their intermittent use. Wearing the same pair of shoes, without socks, often led to an offensive smell. People reported deliberately taking off their shoes and walking barefoot to refresh their feet and shoes:

“Shoes are very important items. The down side of shoes in my opinion is the bad smell they create when worn regularly. You need to have at least two pairs of shoes. Otherwise, if you have only a single pair of shoes, you should not wear them regularly. Putting them aside sometimes helps you to refresh your feet and avoid the bad smell.” (Unaffected male, age 46)

[of the typical plastic shoe locally available] “..one, its price is a bit higher than the sandal type; two, it creates a bad smell during the hot season; and three, its shape is not attractive.” (Unaffected male, age 38)

Financial limitations also dictate the age at which parents begin to provide shoes for their children. Most parents said that they provided shoes for their children when they started school or reached school age, though some respondents suggested that shoes should ideally be provided as soon as children started to walk.

“I have five children. Except one, four of them wear shoes. I buy shoes for them at the age of 8 when they are mature enough to attend school” (Unaffected male, age 48)

“It is only those who are financially strong that can buy shoes for their children at the age of one. Those who have no capacity may not buy shoes even at the age of 10 or 15.” (Unaffected female, age 35)

Two final areas in which financial constraints had impact on use of shoes was in relation to the quality and size of the shoe. There was a clear mismatch between the types of shoes desired (leather shoes and sneakers) and the types of shoes that are available and affordable, which contributed to their inconsistent use. Plastic shoes (locally known as ‘*kongo*’) and foam sandals (locally known as ‘*kitto*’ shoes) are the most prevalent types used in this community. These are cheap but not very durable, and are widely available in the local markets. Although they are accessible, they are the least favored types, afford poor protection and, due to their unattractive designs and the low-quality materials used to make them, may be uncomfortable.

“People who have the capacity wear better shoes like sneakers and leather-made shoes. Most people wear plastic shoes which are low quality and poor strength”. (Affected female, age 42)

“Most of the time I wear *kongo* shoes. I wear these shoes not by preference. *Kongo* shoes are not my preference because they are not durable. I simply buy them because I cannot afford durable shoes which are very expensive for destitute persons like me”. (Unaffected, female, age 35)

“I want to buy leather shoes for my children. That is my preference. However, I am not in a position to do so because of lack of money. I am too poor to cover all the necessities for my family members. Therefore, as a last option I buy them ‘*kitto*’ and plastic shoes. (Unaffected, male age 41)

Patients reported one final barrier: difficulty finding shoes big enough to comfortably fit swollen feet in the local market. The MFTPA was the only source of shoes tailored to the size of patients' feet. Patients who could not access MFTPA shoes often ended up without shoes, as demonstrated in this quote -

“While I was living with my husband I used to wear shoes (‘*kongo*’ plastic shoes). My husband used to buy them for me and my children. I wore out the last pair and threw them away last year. Then, my feet began to swell up. So, I remained barefoot. I can't find shoes that fit my feet at a price I can afford. Now I do not have any type of shoe at all.” (Affected female, age 40)

“I also assume that some patients do not wear shoes just because they can't get a shoe that fits the size of their feet.” (Unaffected male, age 35)

#### ii) Unsuitability of shoes for specific activities

In addition to all the issues related to financial constraints, respondents mentioned practical barriers linked to type of footwear available locally. They reported that the shoes typically owned by farmers were unsuitable for farming activities, meaning that farmers preferred to work barefoot to avoid the discomfort caused by soil and mud entering shoes or sandals:

“I have shoes, but I usually take them off while farming the land. They are not suitable for working, because soil enters the shoes and gives me discomfort. It also carries mud and makes it heavy. Thus, we prefer to work barefoot.” (Unaffected male, age 55)

“We buy those shoes (referring to plastic sandals) at low price … it is not suitable wearing them in the farm, because soil enters into the shoes and gives discomfort” (Unaffected male, age 62)

“We don't use shoes while farming in the wet season or the dry season, because the shoes we have are not appropriate for farming. Soil and mud can easily get into the shoes and make working so difficult.” (Unaffected male, age 55)

“I always wear shoes…[] I came to this place without shoes” (Affected male, age 50)

Low quality plastic shoes are also reported to be slippery on rainy days in muddy places:

“I don't want to wear shoes while walking on muddy places and around the river, because it is impossible to control our balance when wearing shoes” (Unaffected female, age 35)

#### iii) Low perceptions of risk

The remaining barriers to consistent use of shoes were disease specific, and linked either to low perception of risk of podoconiosis or misunderstandings of the causes of this non-infectious, mineral-related condition. Participants who considered themselves to be at low risk of disease were unlikely to wear shoes consistently:

“As my feet are healthy, I don't care about wearing shoes. There are times when I wear shoes, but mostly I prefer to stay barefoot. I wear shoes when I go to church or market, or other places. I sometimes go to such places barefoot.” (Unaffected female, age 30)

“…there is nothing I intentionally do to prevent the disease. I do not worry about it because I know that there is no such disease in the blood line of my family.” (Unaffected male, age 45)

#### iv) Fear of stigma and labeling related to shoes collected from MFTPA

Finally, in this section, patients were also concerned about the design of the shoes distributed to them by the MFTPA. The design differentiates these shoes from typical ‘market’ shoes and may make patients liable to labeling as affected with podoconiosis:

“The shoes we get from the organization exposed us to labeling. So, differently designed shoes are better.” (Affected female, age 55)

In some instances, it was reported that community members have stigmatizing reactions towards patients who are wearing these shoes, even when their foot swelling had resolved. Fear of such reactions discourages consistent shoe wearing:

“Before the treatment, the swelling was larger than you see today. I was also given shoes from the clinic. [Where are they? why don't you wear them?] I wear them most of the time. Today you have seen me playing with friends barefoot just because I am tired of bad insulting words about the shoes. I sometimes hear children commenting on my shoes saying, ‘*kitta* shoes’, meaning a shoe for mossy foot [podoconiosis] patients. I don't like such comments and therefore I take them off while playing around home. I also wanted to show them that my foot is cured and therefore they will not give me such name from now on…I asked my father to buy me different shoes, but he always nags me to wear these ‘*kitta*-shoes’ which I don't like.” (Case study, affected boy, age 13)

### B. Community perceptions favoring footwear

In terms of wearing shoes, it appears that in Wolaita zone, like other rural parts of Ethiopia, people are moving from a ‘norm’ of going barefoot, to one where shoes are worn, and it is becoming ‘shameful’ to appear in public places without wearing shoes. Expansion of schools in rural communities and proliferation of the variety of shoes in local markets have contributed enormously to changing mindsets towards accepting footwear as a valuable commodity:

“… the advancement of education has changed the minds of the people… today appearing barefoot especially in public places affects the dignity of the person.” (Unaffected male, age 45)

The overwhelming majority of respondents were positive about wearing shoes. Both adults and children emphasized that, despite the impediments to securing footwear, everyone in the community was in favor of having shoes:

“…these days, people of any age want to wear shoes”. (Affected female, age 32*)*

The following excerpts demonstrate that social pressures (and not just issues related to disease prevention) are important in driving the community norm towards wearing shoes:

“Here in our community, people give more respect to those wearing shoes. Therefore, to escape the insults, some individuals migrate to town and other locations, stay there doing daily labor and come back wearing shoes.” (Religious leader, male, age 50)

“Educated sons and daughters advise their parents saying ‘people insult me, not you, if you don't wear shoes, I will be ashamed of being your child if you travel barefoot to town’, etc.” (Religious leader, male, age 54)

“Some people would rather wear old and worn out shoes than remain barefoot. Others also strive to buy shoes, borrowing from someone if they do not have money in their pocket, just to be free from insults. When people see a man who does not have shoes they say, ‘is it your leg that hates the shoe or the shoe that hates your leg’?” (Community leader, male, age 45)

Even young children communicate their wish for shoes to parents: attempting to wear their parents' shoes, nagging their parents to buy them shoes, and refusing to attend school barefoot. In many families, it is the children who press their parents into buying their first shoes. As one parent said,

“I buy shoes for my children just for the sake of sending them to school. It is a means of consoling them…” (Unaffected male, age 39)

The following excerpts also illustrate this very well:

“Children also wear shoes. They even try to wear their parents' shoes at home just to demonstrate their interest in wearing shoes. Looking at this, their fathers will buy those shoes …Once the children start wearing shoes, they will keep it up and never want to be in school barefoot. Some children ask their father immediately after joining school in grade one. Then, it will be a must to buy shoes. Otherwise, the child could quit school.” (Religious leader, male, age 50).

“Some of my children, particularly the older ones, are not willing to go to school barefoot and therefore I bought shoes for three of the older children, while the younger children are still barefoot and are not enrolled in school.” (Unaffected, male, age 46)

In this specific area, although the MFTPA has worked to circulate messages about podoconiosis prevention for more than ten years, this does not appear to be an important reason for wearing shoes in the wider community. Adults emphasized using shoes to participate in social settings and public gatherings, while children emphasized the protective value of shoes against pain of walking on stones and other sharp objects. Respondents in many groups also mentioned that shoes protected them from cold and injuries, enabled walking and looked attractive on the feet. In some cases, shoes were worn simply because they saw others wearing them.

“I wear shoes not with the intention to prevent the disease, but because other people wear them.” (Unaffected male, age 38)

Since podoconiosis patients had been advised to wear shoes by the MFTPA, we found that the practices of treated patients differed from those of the general community:

“I was not wearing shoes before my foot became like that. That incident gave me a good lesson and since then, I made my children wear shoes. Otherwise, they may also develop the disease…” (Affected male, age 30)

Among patients, primary prevention may beneficially be linked with disease treatment, and patients using shoes for secondary prevention of complications may not only model behavior changes but also encourage them in children.

“Yes, I do have a pair of shoes that I received from MFTPA. Before treatment, I used to wear shoes only when the temperature became cold, particularly in the morning, because I was afraid of the pain arising from chilly temperature. When the temperature rose, I deliberately took off the shoe and walked barefoot… Lately, after treatment, I realized that this practice was absolutely wrong. I was given a lesson and instructions by the staff that I had to wear my shoes regularly if I want to get a cure. Now, I always wear the shoes given to me by the organization.”(Affected female, age 35)

If I leave home without shoes, I immediately get sick. I can't step even a short distance without shoes. So, shoes are important to protect us from the painful feeling. (Affected female, age 28)

## Discussion

This study, which aimed to explore shoe wearing practices in a podoconiosis-endemic setting in rural Ethiopia, brings to light several issues relevant to other foot-related NTDs. We discovered that, despite a clear wish to wear shoes, and to wear them regularly, many practical and social barriers prevent these wishes being translated into practice. Many of the barriers cited will be relevant to those considering distribution of shoes to prevent snakebite, tetanus or helminthiases. We also witnessed inconsistency between reported and actual shoe wearing behavior, confirming the complexities that exist in relation to recording shoe use. We suspect that these complexities may not have been adequately addressed in earlier studies on risk factors for a range of NTDs.

### Who is wearing shoes, and why?

Shoe wearing was intermittent, with adults more likely to say they wore shoes for social events and gatherings including market attendance, church services, weddings and funerals. Farmers, both male and female, rarely wore shoes while working in the fields, and many householders did not wear them while gathering wood or fetching water. Although children were usually encouraged to wear shoes at school, they were often dissuaded, sometimes forcibly, from wearing them for housework or play.

More consistent use of shoes was reported by podoconiosis patients than the wider community, several patients referring to advice received from the MFTPA. Perception of risk appeared to be an important contributor to this difference in behavior: patients reported changing their own shoe wearing behavior and influencing that of their children, while non-affected community members wore shoes less or not at all. Several articles have linked risk perception with actions related to health-seeking behavior, people with higher perceived vulnerability to illness being more likely to engage in protective behavior [30]. Research on foot care and footwear practices of peoples with diabetes [31], [32] has demonstrated similar links between use of shoes and perceived risk of disease to those presented here. This suggests that any future NTD interventions based on shoe distribution to individuals with disease must be accompanied by messages that appropriately convey mechanisms by which diseases occur and individual and community levels of risk.

While patients viewed shoes as a means of protection from disease, non-affected adults indicated they were beginning to have more general social value. Shoe wearing was seen as a mark of dignity, while going barefoot was seen as ‘shameful’, particularly by the younger generations. Some participants suggested that shoe wearing norms were in the process of change, and one directly ascribed this to education - “the advancement of education has changed the minds of the people… today”. Clearly, drivers of change in this norm are acting at many levels, and though some may be harnessed in intervention programs, others will be beyond easy reach. Children are also aware of the ‘shame’ of going to school barefoot, but also mentioned the role of shoes in preventing injuries from stones, thorns and other sharp objects. Future programs will need to address all these motivations for shoe use and highlight the range of benefits that shoe wearing is likely to bring.

### Barriers to shoe wearing

Recurring barriers mentioned by study participants that are likely to be relevant in other NTD-endemic communities, were those of financial constraint and poor suitability of shoes for the most common activities. Financial constraints were reported to influence possession of shoes, type of shoe bought, age at which a child starts wearing, which children get shoes within families, consistency of use, frequency of replacement and activities for which they were worn. As with many health interventions whose benefits will only become apparent in the longer term, families naturally prioritized more immediate concerns such as food. Currently, in this area, shoes are being distributed free of charge, but this is unlikely to be sustainable in the long term or scalable to all rural populations exposed to NTDs. However, if shoes are to be considered health interventions rather than pure commodities, subsidies or micro-credit strategies that bring shoes within the reach of very-low income families must be contemplated. Social protection strategies like these may bring the necessary empowerment for individuals to realize the behavior changes they may desire to make.

Several participants gave highly practical reasons for preferring not to wear shoes while farming, saying that the shoes available in the market quickly became heavy with mud and failed to grip in the rainy season, and became uncomfortable when rough soil particles slipped inside during the dry season. Long Wellington-type boots might prevent these problems, but are more expensive than the shoes currently available. Clearly, promoting footwear that is appropriate to local activities and effective against the specific NTD is essential. For example, the prevention of snakebites in rice paddies will require different footwear than those required for the prevention of chronic larva migrans on the beach.

Some barriers to use of footwear were patient-specific. Swelling of the feet and lower limbs, nodules and wounds may make use of normal-shaped shoes impossible. Molla and colleagues [33] have documented similar challenges faced by podoconiosis patients in northern Ethiopia. Custom-made shoes might overcome these difficulties, and have been developed for patients with leprosy in similar resource-limited rural communities. Legs to Stand On, an initiative to prevent disabling disease of the lower limb in resource-poor settings, is leading cross-disease efforts to increase capacity to manufacture custom-made shoes in these communities.

However, custom-made shoes bring with them the possibility of stigma through labeling as ‘diseased’. This was raised as a very real barrier by a number of patient participants in our study. Some had developed tactics to mitigate the stigma they faced, by removing their shoes in certain situations, while others had abandoned them completely. Stigma has been documented, against podoconiosis patients and their families [26], [34] against patients with leprosy [35] and lymphatic filariasis [36], and interventions against these or other NTDs must not risk increasing stigma. In the future, much more attention must be directed to the design of custom-made shoes so that they do not increase stigma in relation to any NTD.

### Implications for measurement of shoe wearing behavior

Several investigators have suggested that lack of association between footwear and disease in observational studies reflects poorly refined measurement of shoe wearing behavior. In most studies, there is no clear definition of the length of time spent wearing shoes or the activities for which they are worn. For example, while individuals may state they wear shoes ‘most of the time’, they may remove them to plough, sow, harvest or fish. These activities may represent the time of greatest exposure to infective or other agents. Many quantitative studies investigating the link between incidence or prevalence of NTDs and footwear have used simple questions such as ‘Do you wear shoes?’ with binary response options [8], [11], [13]–[15]. Our participants describe complex behaviors, wearing shoes in certain settings (including in church services and at school) but not in others (often those where exposure is more likely, such as farming). Clearly, more nuanced questions must be asked of study participants if a true picture of shoe wearing is to be captured. Better designed questions, informed by qualitative research, will allow identification of potential points of behavioral intervention that take into account the structural barriers posed by rural poverty.

### Conclusions

We have explored behaviors related to use of shoes in a low-income rural setting where several NTDs including podoconiosis are prevalent [37]. We used a range of qualitative techniques among multiple target groups. Although the study included a large sample, all respondents were drawn from the same rural highland community in Ethiopia, and so we suggest caution in generalizing the reported outcomes to other cultural settings. Although we hoped to reduce social desirability bias by collecting data through individuals not linked to the MFTPA organization, it is likely that the information given by some respondents was still influenced by their wishes for perceived social conformity.

Although shoes are desired, they are either not worn or not worn sufficiently consistently to prevent disease. Consistent with well established conceptual models of health behavior adoption, we identified several barriers to shoe wearing that are amenable to intervention [25]. Moreover, several of these barriers will arise in other settings in relation to other NTDs, and we encourage program developers to consider each of these before developing theory-based interventions to encourage shoe wearing.
